# Emergent Cardiac Surgery After Transcatheter Structural Heart Procedures: Narrative Review

**DOI:** 10.1002/ccd.31519

**Published:** 2025-04-07

**Authors:** Miriam Compagnone, Gianni Dall'Ara, Simone Grotti, Daniela Spartà, Giuseppe Guerrieri, Carmine Pizzi, Fabio Felice Tarantino, Marcello Galvani

**Affiliations:** ^1^ Interventional and Structural Cardiovascular Unit, Forlì‐Cesena AUSL Romagna Italy; ^2^ Department of Medical and Surgical Sciences—DIMEC—Alma Mater Studiorum University of Bologna Bologna Italy; ^3^ Cardiology Unit Bufalini Hospital Cesena Cesena Italy; ^4^ Cardiology Unit Morgagni Pierantoni Hospital Forlì Forli Italy; ^5^ Cardiovascular Research Unit Fondazione Cardiologica Sacco Forlì Italy

**Keywords:** emergent cardiac surgery, left atrial appendage occlusion, transcatheter aortic valve replacement, transcatheter edge‐to‐edge repair

## Abstract

Transcatheter structural heart procedures have become standard therapy for elderly patients with high surgical risk. Over time, these procedures have significantly increased worldwide, accompanied by a concomitant reduction of major complications, including those requiring emergent cardiac surgery (ECS). This marked decline in ECS is due to technological advancements, improved patient selection and procedural techniques, and increased institutional and operators expertize. Moreover, most major structural complications after transcatheter structural heart procedures are now managed percutaneously, with only a small proportion requiring ECS. It is important to note that outcomes for patients requiring ECS remain unfavorable, even in the optimal setting. Currently, ECS after percutaneous structural interventions is very rare, less than 0.5%, as reported in multicenter available studies. However, fragmented data exist in the literature on the need of ECS. Indeed, low incidence, different definitions, and lack of recent reports make it difficult to have a precise and up‐to‐date overview of bailout surgery for treatment of procedural complications. This is the first comprehensive analysis focusing on ECS following the major frequent percutaneous structural procedures, that is, transcatheter aortic valve replacement, mitral valve repair/replacement, and left atrial appendage occlusion. More in general, a collaborative approach among Heart Team members, along with thorough procedural planning guided by advanced imaging techniques, is essential for ensuring high‐quality interventions thus minimizing the risk of adverse events.

**Abbreviations**
ECS: emergent cardiac surgery;
LAAO: left atrial appendage occlusion;
TAVR: transcatheter aortic valve replacement;
TEER: transcatheter edge‐to‐edge repair;
TMVR: transcatheter mitral valve replacement;
US: United States

## 1 Introduction

A percutaneous structural procedure refers to a minimally invasive intervention performed via catheter‐based techniques to treat structural heart diseases. These procedures are typically performed in a catheterization laboratory, specifically designed for rapid conversion in open surgery (hybrid operating room). These interventions include a range of treatments for valvular heart disease, such as transcatheter aortic valve replacement (TAVR) and transcatheter edge‐to‐edge repair (TEER) of the mitral valve, and left atrial appendage occlusion (LAAO) for stroke prevention. They also encompass interventions for congenital heart defects, such as patent foramen ovale and atrial or ventricular septal defect closures, as well as percutaneous treatment of paravalvular leaks. These interventions, due to the low frequency of complications or paucity of data, are excluded from this discussion.

**Figure 1:**
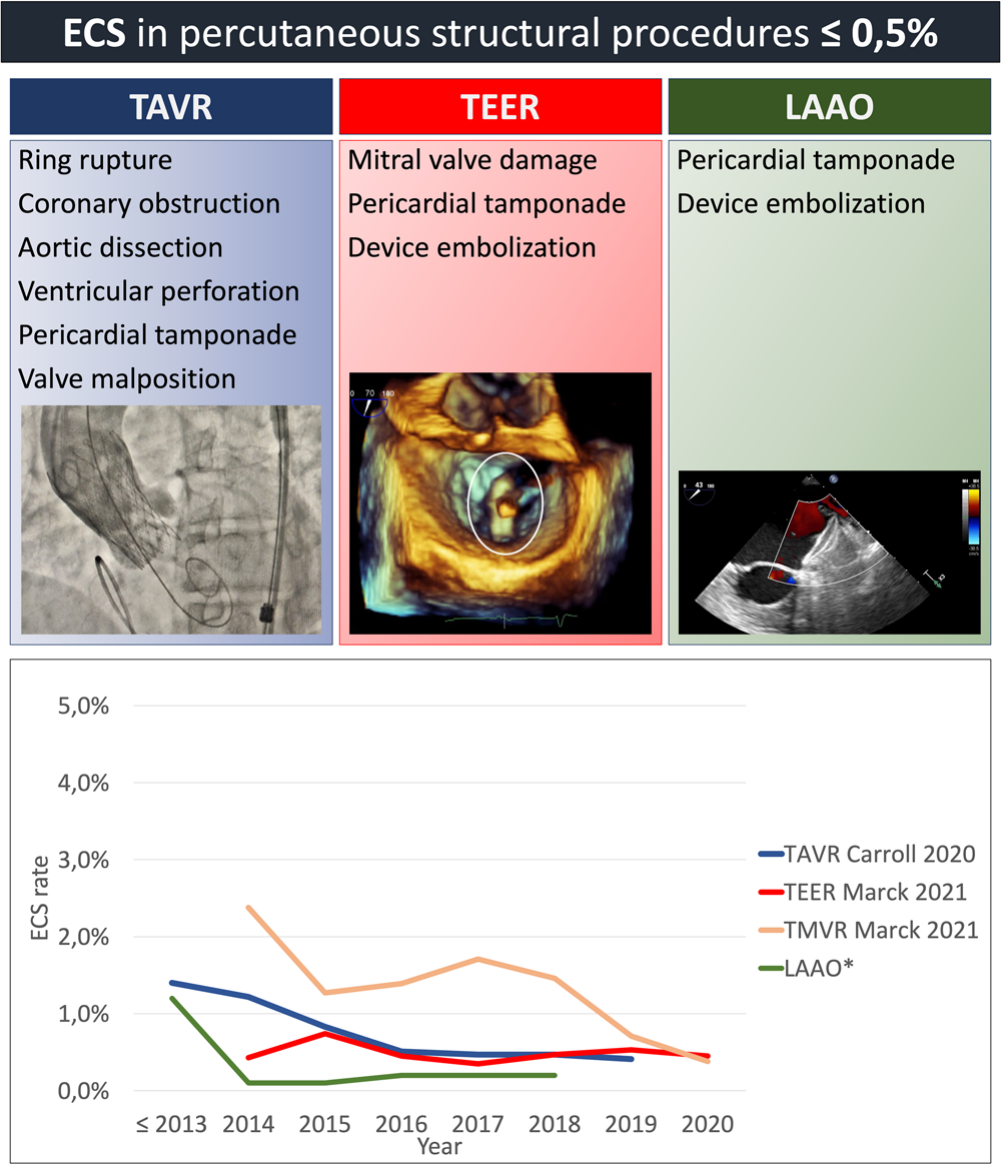
Complications potentially requiring ECS and there rate in principal percutaneous structural procedure studies from 2013 to 2020. ECS = emergent cardiac surgery; LAAO = left atrial appendage occlusion; TAVR = transcatheter aortic valve replacement; TEER = transcatheter edge‐to‐edge repair; TMVR = transcatheter mitral valve replacement. *Date include both ECS due to life‐threatening complications and subsequent need of mitral valve replacement or repair. †Reference [14, 19, 20]. [Color figure can be viewed at wileyonlinelibrary.com]

Percutaneous structural procedure offer an alternative to traditional open‐heart surgery and are particularly beneficial for patients who are at high risk for surgery due to important comorbidities. Over the last two decades various randomized clinical trials have demonstrated the efficacy of percutaneous treatment compared to medical therapy [1, 2, 3, 4], and the non‐inferiority compared to surgical treatment in specific settings [5] and different class of surgical risk [6, 7, 8, 9, 10, 11]. Observational data from the United States (US) show that there has been a rapid increase in percutaneous structural procedures over time and, accordingly, a gradual decline of patients' risk profile [12, 13, 14]. In fact, nowadays, interventional therapy is no longer reserved for patients with high surgical risk, since an increasing number of intermediate‐ and low‐risk patients with structural heart disease are being treated with interventional procedures. These data, together with the constant technological improvement of the devices, the reduction of complications, and the encouraging long‐term durability, suggest that transcatheter procedures are becoming the principal treatment of several heart diseases. The choice between surgical and transcatheter interventions must be based upon careful evaluation of clinical, anatomical, and procedural elements by the Heart Team, weighting risks, and benefits of each approach for an individual patient. Current international guidelines affirm the importance to perform transcatheter heart valve procedures in Heart Valve Centers with on‐site cardiac surgery, especially in the case of TAVR (class of recommendation I, level of evidence C) [15, 16]. The simultaneous presence of different medical specialties is considered the optimal condition to ensure appropriate patient selection, effective treatment, as well as prompt management of potentially severe structural complications, possibly requiring emergency cardiac surgery (ECS). In particular, younger and lower‐risk patients are those with the highest likelihood to survive major complications requiring ECS, which is guaranteed by the presence of on‐site cardiac surgery. ECS is generally defined as any cardiothoracic surgical intervention in bailout after transcatheter procedures, including urgent valve replacement, removal of embolized device, repair of myocardial or aortic injury, and coronary artery bypass [17, 18]. Fortunately, severe structural complications are exceedingly rare and, as a matter of fact, infrequently addressed surgically, as shown in the Central Figure 1.

## 2 ECS Post‐TAVR

In pivotal TAVR randomized trials the incidence of ECS was not specified. Analyzing the results of the PARTNER trial cohort B, among 179 patients randomized to TAVR, 1 (0.6%) had valve embolization, and 2 (1.1%) underwent multiple (≥ 2) valve implantations. No conversion to open surgery was observed for the management of these complications [1]. In cohort A, the transcatheter procedure was either aborted or converted to open surgery in 16 of 348 patients (4.6%), as a result of new intraprocedural findings, failure to obtain femoral access, or procedural complications. Among these 16 cases, 9 underwent open surgery immediately (including one who died) [6]. In the CoreValve trial, five cases of left ventricular perforation occurred (1.3%) [7]. In the intermediate‐risk PARTER trial, there were three cases of conversion to cardiac surgery due to valve embolization (0.3%) [8]. Table 1 shows the percentage of major structural complications and conversion rate to cardiac surgery in the main international TAVR registries [12, 21, 22, 23, 24, 25, 26, 27, 28]. These data are derived from studies covering an enrollment period from 2007 to 2023, from the dawn of the TAVR procedure performed with first‐generation devices to recent years where third‐ or fourth‐generation prosthesis are used. In parallel with device improvement, the acknowledgment of the importance of procedural planning has greatly changed the technical approach and prosthesis choice. Over time, the studies have progressively included younger patients with lower surgical risk, and femoral access has become the most commonly used approach. As a result, the observed decline in procedural complications and the need for ECS is unsurprising. In addition, the timing of surgical interventions was not always specified. Consequently, the incidence of ECS after TAVR could be lower, as shown in the SOURCE registry, where the rate of conversion to open surgery was 2.7% while the risk of ECS was 1.2% [21, 29]. In two meta‐analyses published in 2013, the probability of ECS after TAVR was 1.1% and 1.2% respectively; the rate was lower in case of trans‐arterial than in case of trans‐apical approach (0.6% vs. 2% respectively) [30, 31]. In the European multicenter study of 27760 transfemoral TAVR procedures, performed between 2013 and 2016, ECS was necessary in 212 patients (0.8%) and the most common causes were left ventricular perforation by the guidewire in 60 (28.3%), annular rupture in 45 (21.2%), and device embolization in 27 patients (12.7%) [26]. Nowadays, not all major structural complications are treated by surgical intervention. In the TRAVEL registry there were 273 (0.9%) cases of peri‐procedural transcatheter valve embolization and migration. Two‐hundred twenty‐one cases (81%) were managed percutaneously (repositioning or/and second valve implantation); only 52 patients (19%) required open surgery [32]. The use of self‐expanding or first‐generation prostheses and presence of a bicuspid aortic valve were independent predictors of transcatheter valve embolization and migration [32]. Ribeiro et al. showed that most cases (approximately 75%) of acute coronary obstructions after TAVR can be treated by percutaneous coronary intervention (success rate 82%) [33]. In particular, the authors observed that the lower position of the coronary ostium and a shallow sinus of Valsalva are significant anatomic factors associated with this complication. Despite successful treatment, both early and late mortality remain notably high, emphasizing the critical importance of anticipating and preventing the occurrence of these complications [33]. Indeed, the routine use of imaging techniques to establish the procedural strategy reduces the risk of the most dangerous adverse events through better selection of patients to TAVR and better choice of the correct valve type and size. In a multicenter study by Barbanti et al., which reported 31 cases of aortic root rupture after balloon‐expandable valve implantation, only 12 patients (39%) were treated with open surgery, of which 9 patients died (75%). A total of 15 patients were treated conservatively with 5 deaths (33%). The overall mortality rate was 48%, with 75% mortality in cases of uncontained rupture [34]. The two key features associated with annular rupture or periaortic hematoma are: moderate to severe sub‐annular calcification and prosthesis oversizing ≥ 20%, as assessed by pre‐TAVR computed tomography analysis [34]. Therefore, the prevention of complications needing ECS remains the most important strategy for improving outcome. In the STS‐ACC registry, that analyzed a population of 276316 TAVR patients, ECS decreased from 1.4% before 2013 to 0.4% in 2019 [12]. The reduction of these events was related to increased operator expertize, less frequent use of the trans‐apical access, procedural planning with routine use of imaging techniques, and rapid development of new devices. In a recent study with new generation balloon expandable transcatheter valve (SAPIEN 3 Ultra Resilia) in native TAVR patients conversion to open surgery was 0.2%. There were 21 annular rupture/aortic dissections, only 3 treated with ECS; 45 ventricular perforations/tamponades, treated with ECS in only 4 cases; and 9 coronary obstructions, treated with ECS in only one case [28].

**Table 1:** Conversion to open surgery and in hospital main structural complication after TAVR in principal international registries, according to the time of enrollment [12, 21, 22, 23, 24, 25, 26, 27, 28].

	Enrollment	N.° of patients	Mean age	LogES/STS (%)	Transfemoral approach (%)	Conversion to surgery (%)	Ring rupture(%)	Aortic dissection (%)	Coronary obstruction (%)	Cardiac tamponade (%)	Valve malposition (%)	Ventricular perforation (%)	2° Valve implantation (%)
Source Thomas et al.	2007−2008	1038	81	27/NA	45	2.7	NA	NA	NA	NA	0.3	NA	NA
France 2 Auffret et al.	2010−2012	4165	83	22/NA	73	1.2	0.3	0.2	NA	1.3	1.3	NA	2.3
Gary Walther et al.	2010−2015	15,964	81	18/5	71	1.3	0.4	0.2	NA	1.0	NA	NA	NA
STS‐ACC TVT Pineda et al.	2010−2015	47,546	83	NA/7	72	1.2	0.2	0.1	0.07	NA	0.3	0.2	NA
France Auffret et al.	2013−2015	12,557	83	18/NA	83	0.5	0.4	0.4	NA	2	1.1	NA	1.8
Source 3 Wendler et al.	2014−2015	1947	81	18/NA	87	0.6	0.2	NA	0.4	NA	NA	NA	0.7
EuRECS‐TAVI Eggebrecht et al.	2013−2016	27,760	NA	NA	100	0.8	0.2	0.1	0.04	0.05	0.1	0.3	NA
STS‐ACC TVT Carroll et al.	2011−2019	276,316	81	NA/5	90	0.6	NA	NA	NA	NA	NA	NA	NA
CENTER2 Aarts et al.	2007−2022	24,010	82	14/5	100	0.5	0.1	0.04	0.02	NA	0.1	0.2	NA
STS‐ACC TVT‐S3UR Stinis et al.	2021−2023	10,314	77	NA/4	97	0.2	0.2	0.1	0.1	NA	0.1	NA	NA

## 3 What Is the Outcome After ECS Post‐TAVR?

Although rare, complications requiring ECS are associated with high mortality. As reported in the SOURCE registry, patients undergoing surgery after TAVR had a mortality rate of 52% at 30 days, reaching 100% in case of aortic ring rupture [21]. Eggebrecht et al. analyzed the outcomes of 212 patients requiring ECS. Most complications (>90%) occurred and manifested acutely during TAVR procedure. The mortality rate was 33% within 72 h from ECS, 46% in‐hospital and 78% at 1 year. Patients with annular rupture were at the highest risk with an in‐hospital mortality rate of 62% [26]. To date, the largest study reporting the need of ECS during TAVR included 47546 patients who underwent TAVR between the years 2011–2015 in the US. ECS was performed in 558 (1.2%) cases and, as expected, was associated with a very high in‐hospital and 1‐year mortality (50% and 60%, respectively) [24]. Recent data reported that ECS occurred in 0.52% of patients, and its incidence decreased over time, from 0.84% to 0.25%. Procedural mortality frequently observed in ECS patients compared with those without surgical bailout (25.6% vs 1.1%), being in‐hospital mortality (48.0% vs 3.5% respectively) [27].

In a large tertiary center experience, ECS outcome changed according to patient surgical risk: in hospital mortality was 62.1% in high‐risk patients and 12.5% in low/intermediate risk patients [35].

In conclusion, adverse events after TAVR requiring ECS are often life‐threatening because associated with sudden hemodynamic collapse with a poor prognosis irrespective of surgical treatment, especially in patients at high surgical risk.

### 3.1 ECS After Transcatheter Mitral Valve Repair/Replacement and Its Outcome

According to data available in the literature, over 150,000 patients have been treated with TEER worldwide [36]. This figure reflects the cumulative experience developed with the MitraClip (Abbott Vascular, Santa Clara, California) device for the treatment of mitral regurgitation, particularly in patients who are at high or prohibitive risk for surgery [15, 16]. According to the consensus document from the Mitral Valve Academic Research Consortium [18], conversion to mitral valve surgery during a transcatheter procedure is subclassified as secondary to mitral valve apparatus damage or dysfunction, or secondary to procedural complications such as cardiac perforation, removal of an embolized device, etc. As reported in the EVEREST II randomized trial, no emergent conversion to open surgery for adverse events was observed in the percutaneous group [5]. In the COAPT study only one case required ECS due to post‐procedural pericardial tamponade: following successful pericardiocentesis, surgery was performed because of concerns of ongoing bleeding, but during surgical inspection no perforation was found [2]. In MITRA‐FR two patients suffered from cardiac tamponade that occurred during trans‐septal puncture, while no cases of ECS were observed [37]. Real world data from STS database, showed that from 2014 through 2020 a total of 33878 patients underwent TEER with the MitraClip system in US and in‐hospital conversion to open heart surgery occurred in 0.5% of case [13] but, notably, only in 0.08% of cases in emergency [38]. In general, in TEER, conversion to surgery was very rarely a life‐saving procedure but most often performed for ineffective reduction of mitral regurgitation. The conversion rate to cardiac surgery after TEER in the principal international registries is reported in Table 2 [13, 39, 40, 41, 42, 43, 44]. The population of these studies is very heterogenous, including both functional and degenerative mitral regurgitation. In a retrospective study, among 15,032 eligible TEER patients, ECS was required in 214 (1.4%), being however necessary the same day of TEER in less than 50% of patients. The incidence of ECS decreased significantly over the 4 years of observation (5.26% in 2014; 0.43% in 2017; *p* < 0.001), and was higher in hospitals with lower annual volume (less than 18 cases per year). Cardiac surgery after TEER was significantly associated with a higher rate of in‐hospital adverse events; in particular all‐cause mortality was 15% [45]. Actually, the definitions of “conversion to open surgery” may vary between different studies, often including non‐ECS due to unsatisfying procedural results.

**Table 2:** Conversion to open surgery and in hospital main procedural complications after TEER in principal international registries, according to the time of enrollment [13, 39, 40, 41, 42, 43, 44].

	Enrollment	N.° of patients	Mean age	LogES/STS score (%)	Conversion to surgery (%)	Serious pericardial effusion (%)	Device embolization (%)	Single leaflet device attachment (%)
ACCESS‐EU Maisano et al.	2009−2011	567	74	23/NA	1.1	0.9	0.0	4.8
Pilot European Sentinel Registry Nickenig et al.	2011−2012	628	74	20.4/NA	NA	1.1	0.7	NA
GRASP Attizzani et al.	2008−2013	171	72	NA/6.5	0.0	0.0	NA	0.0
TRAMI Puls et al.	2010−2013	828	76	20/6	0.8	1.7	0.0	0.7
MitraSwiss Surder et al.	2011−2018	1265	79	7.2/3.7	0.9	NA	NA	NA
STS‐ACC TVT Mack et al.	2014−2020	33,878	80	NA/5.4	0.5	NA	NA	1.0
MiCLASP Lurz et al.	Published in April 2024	544	77	NA/5.1	0.4	NA	NA	0.2

The PASCAL (Edwards Lifesciences, Irvine, California) transcatheter valve repair system has been shown to be a safe and effective alternative to the MitraClip device, with comparable technical success rates and clinical outcomes. In the CLAPS study only one patient (1.6%) had a single‐leaflet device attachment. The procedure was converted to surgical mitral valve replacement, but timing (emergent vs. elective) was not specified [46]. In the CLAPS IID trial, the randomized comparison between PASCAL and MitraClip system, ECS after TEER did not occur. However, in the PASCAL group one patient had a single‐leaflet device attachment and underwent mitral valve percutaneous reintervention with an additional PASCAL device, while in the MitralClip group there was an irrecoverable entrapment of the device in the sub‐valvular chordal apparatus resulting in death [47]. In the most recent prospective and single arm MiCLASP Study, conversion to cardiac surgery was necessary in 2 out of 544 patients (0.4%) treated with the PASCAL device. One case was due to inadequate mitral regurgitation treatment and the other one to high gradient after leaflet grasping [44].

The transcatheter mitral valve replacement (TMVR) is developing as an alternative to surgery for patients with severe mitral valve disease due to degenerated mitral bioprostheses, failed surgical repairs with annuloplasty rings, or native mitral valve disease with severe mitral annular calcification (MAC), a condition associated with poor early outcome due to the presence of high calcium burden that increase the technical challenges. Data from a retrospective analysis, including 903 TMVR patients, showed that the rate of ECS was 2% in case of MAC (*N* = 100 patients, 11%). The risk of left ventricular outflow obstruction and need for a second valve were also very high in this contest, 10% and 14% respectively [48].

TMVR rate of complications has also decrease overtime. The STS registry included a total of 3597 patients and reported that conversion to open heart surgery occurred in 43 patients (1.2%). The incidence dropped from 2.4% in 2014 to 0.4% in 2020, likely associated with an important shift in procedural approach, from predominantly transapical (76% of cases in 2014) to predominantly transseptal access (83% in the first quarter of 2020). This transition to transseptal access was associated with a year‐by‐year decline in adverse short‐term outcomes and an increasing percentage of patients alive at 1‐year [13].

### 3.2 ECS After LAAO

Actually, the incidence of cardiac surgery conversion during LAAO procedures is relatively low. According to the SCAI/HRS Expert Consensus Statement the rate of serious complications is expected to be less than 2% [49]. A surgical intervention may be required due to device embolization or pericardial effusion leading to tamponade, which may be caused by perforation of the left atrial appendage wall or damage to other cardiac structures during transeptal puncture.

In the PROTECT‐AF trial there were 3 cases of device embolization (0.6%), of which only one (0.2%) underwent ECS because the Watchman device was entrapped in the left ventricle outflow tract [3]. The incidence of serious pericardial effusion (i.e., requiring percutaneous or surgical drainage) was 4.8% (*n* = 22 of 463), resulting in seven patients treated with surgical intervention (1.5%) and 15 with pericardial drainage (3.2%) [3]. In the continued access Registry the rate of serious pericardial effusion was less than half that seen in PROTECT AF, with a relative reduction of 58% (*p* = 0.014) [19]. In the PREVAIL trial the incidence of pericardial effusions requiring surgical repair was 0.4% and the device embolization rate was 0.7% [4]. Both trials (PROTECT‐AF and PREVAIL) represent the earliest LAAO experience. As expected, in subsequent studies there was a marked reduction of the periprocedural complications rate (Table 3) [3, 4, 14, 19, 20, 50, 51]. In the EWOLUTION registry, among 1025 subjects, serious pericardial effusion occurred in five cases (0.05%), device embolization in two patients (0.02%), requiring surgically removal of the device in one (0.01%) [20]. In the NCDR LAAO Registry 38158 patients have been treated with the commercial Watchman (Boston Scientific) device from 2016 to 2018. Serious pericardial effusion was documented in 528 (1.4%) patients, of which 91 required ECS (0.2%). Device embolization occurred in 0.07% of the overall cohort [14]. In a prospective observational study with the Amplatzer and Amulet (Abbott Vascular, Santa Clara, California) devices the rate of ECS was 0.2% (2/1088 patients). One case was due to left atrial appendage ostium perforation treated with surgical pericardial patch and the other was related to device embolization entangling the mitral valve [50]. In the Amulet IDE trial pericardial effusion rate was 2.4% (22/903 patients) in the Amplatzer arm and 1.2% (11/896 patients) in the Watchman group. Device embolization was 0.7% (6/903 patients) and 0.2% (2/896 patients), respectively [52]. ECS was unnecessary in any case [52].

**Table 3:** Conversion to open surgery and in hospital major procedural complications after LAAO in principal studies, according to the time of enrollment [3, 4, 14, 19, 20, 50, 51].

	Enrollment	N.° of patients	Mean age	CHADS2/CHA2DS2 score	Conversion to surgery (%)	Serious pericardial effusion (%)	Device embolization (%)
PROTECT AF Holmes et al.	2005−2008	463	71	2.2/3.4	1.5	4.8	0.7
CAP Reddy et al.	2008−2010	460	74	2.4/NA	1.2	2.2	0.0
PREVAIL Holmes et al.	2011−2013	269	74	2.6/3.8	0.4	1.8	0.7
EWOLUTION Boersma et al.	2013−2015	1025	73	2.3/4.5	0.1	0.5	0.2
Amulet registry Hildick‐Smitht et al	2013−2015	1088	75	NA/4.2	0.2	1.4	0.2
NCDR Freeman et al.	2016−2018	38,158	76	NA/4.6	0.2	1.4	0.07
EMERGE LAA Alkhouli et al.	2021−2022	5499	77	NA/4.7	0.2	1.3	0.2

The recent EMERGE LAA study, 5499 patients were treated with Amulet occluder device. Serious pericardial effusion and device embolization requiring ECS occurred in 8 (0.15%) and 3 (0.05%) patients, respectively. The rate of any in‐hospital major adverse events was higher for less experienced operators (less than 10 cases) than experts (more than 30 cases), primarily driven by the incidence of serious pericardial effusions [51].

In summary, pericardial effusion is the most common serious LAAO complication; the majority of cases can be managed percutaneously, only a few necessitating ECS. Price et al. analyzing the clinical course of 881 procedures complicated by serious pericardial effusion (1.4% of 65,355 patients enrolled in NCDC LAAO registry), found that ECS was necessary in 177 cases (0.3% of the entire population). These patients were at significantly higher risk for adverse in hospital outcome and, in particular, the mortality rate of patients requiring ECS was 11.9%, compared to 4.7% in patients who underwent percutaneous pericardiocentesis [53].

In a prospective study including 1023 consecutive LAAO procedures, serious pericardial effusion occurred in 24 (2.3%) patients, of which the majority during the first 24 h after the procedure. ECS was required in five cases (0.5%) [54].

As previously discussed, device embolization is an uncommon complication. In a study cohort of 103 cases, device embolization was more frequently detected in the postoperative period (40 cases) and associated with a higher risk of serious complications and mortality compared with embolization detected intraoperatively (63 cases). This finding is likely explained by the fact that the most common site of intraoperative embolization is the left atrium, with 94% of cases treated percutaneously by a snare. Conversely, postoperative device embolization occurred more commonly to the left ventricle and more likely required surgical intervention compared to other sites [55]. Of 120,278 Watchman procedures, device embolization or migration occurred in 84 patients (0.07%) during the index hospitalization and ECS was performed in 49 patients (0.04%). This complication was more common with the first‐generation Watchman device. In‐hospital mortality rate was 14% among patients with device embolization and 20.5% among patients who underwent ECS [56].

## 4 Conclusions

Nowadays, ECS after percutaneous structural interventions is very rare. As reported in multicenter registries there has been a reduced rate of structural procedure complications requiring surgery, currently less than 0.5% at least in centers with great experience and high procedural volume. The reduction of major complications is due to technological improvements, better patient selection, advancements in procedural techniques, and growing institutional and operator experience. Furthermore, only a minority of major complications is treated with cardiac surgery, being the outcome of these patients often unfavorable, even in the optimal setting comprising on‐site cardiac surgery. More in general, the decision to perform ECS is based on patients age, hemodynamic status, comorbidity and type of complications. While some complication, such as annular rupture during TAVR, are almost always associated with poor surgical outcomes, others, such as LAA perforation that does not stabilize after pericardiocentesis, can have a favorable outcome when managed surgically. This nuanced perspective highlights the importance of having cardiac surgery available for specific complications, despite the low incidence of ECS. Therefore, the ability to respond promptly to complications requiring ECS is crucial for patient safety. The high expertize of the operators, along with close cooperation among all members of the Heart Team, are essential to ensuring high‐quality interventions and minimizing the risk of adverse events. Currently, TAVR is recommended to be performed in centers with on‐site cardiac surgery, whereas LAAO and TEER do not share the same indication. In these procedures, in case of complications likely to be dealt with cardiac surgery, a prompt transfer must be guaranteed to the patient to the hub center with available surgery.

In the case of TAVR, it is worth mentioning that ongoing randomized and prospective observational studies are evaluating the feasibility and safety of the procedure in centers without CS on‐site but where skilled TAVR operators work in a setting with vascular surgery and third level intensive cardiac care unit. These research programs are currently targeting high and prohibitive surgical risk patients [57, 58].

The implication of this finding is the potential to expand transcatheter structural procedures to qualified centers without on‐site CS, but with a well‐established network for proper patients' evaluation and timely management of ECS in the Heart Valve Center.

## Conflicts of Interest

The authors declare no conflicts of interest.
